# Bioenergetics and the Role of Soluble Cytochromes *c* for Alkaline Adaptation in Gram-Negative Alkaliphilic *Pseudomonas*

**DOI:** 10.1155/2015/847945

**Published:** 2015-02-02

**Authors:** T. Matsuno, I. Yumoto

**Affiliations:** ^1^Laboratory of Environmental Microbiology, Graduate School of Agriculture, Hokkaido University, Kita-ku, Sapporo 060-8589, Japan; ^2^Bioproduction Research Institute, National Institute of Advanced Industrial Science and Technology (AIST), 2-17-2-1 Tsukisamu-Higashi Toyohira-ku, Sapporo 062-8517, Japan

## Abstract

Very few studies have been conducted on alkaline adaptation of Gram-negative alkaliphiles. The reversed difference of H^+^ concentration across the membrane will make energy production considerably difficult for Gram-negative as well as Gram-positive bacteria. Cells of the alkaliphilic Gram-negative bacterium *Pseudomonas alcaliphila* AL15-21^T^ grown at pH 10 under low-aeration intensity have a soluble cytochrome *c* content that is 3.6-fold higher than that of the cells grown at pH 7 under high-aeration intensity. Cytochrome *c*-552 content was higher (64% in all soluble cytochromes *c*) than those of cytochrome *c*-554 and cytochrome *c*-551. In the cytochrome *c*-552-dificient mutant grown at pH 10 under low-aeration intensity showed a marked decrease in *μ*_max⁡_ [h^−1^] (40%) and maximum cell turbidity (25%) relative to those of the wild type. Considering the high electron-retaining abilities of the three soluble cytochromes *c*, the deteriorations in the growth of the cytochrome *c*-552-deficient mutant could be caused by the soluble cytochromes *c* acting as electron storages in the periplasmic space of the bacterium. These electron-retaining cytochromes *c* may play a role as electron and H^+^ condenser, which facilitate terminal oxidation at high pH under air-limited conditions, which is difficult to respire owing to less oxygen and less H^+^.

## 1. Introduction

Microorganisms are widely distributed in various environments in nature, such as in cold regions in Antarctica in areas of high temperature and pressure such as in black smoker chimneys in the deep sea and in environments with high osmotic pressure such as the Dead Sea. These microorganisms living in extreme environments are called extremophiles [1]. Extremophiles have provided us with enzymes that can work under corresponding growth conditions [2]. Among such extremophiles, there are microorganisms living at high pH such as above pH 10 [3–9]. Alkaliphilic* Bacillus* species have been isolated for the investigation of their physiological function of adaptation to high pHs and for the utilization of their enzymes [3]. While most extremophiles are living in specific environments, for example, psychrophiles are living in permanently cold environments such as the polar regions, alkaliphiles, that is, alkaliphilic* Bacillus *species, are wildly distributed not only in specific environments related to alkaline conditions, such as alkaline soda lakes, indigo fermentation fluid, and the intestinal tracts of termites, but also in environments not related to alkaline conditions, such as ordinary soil, deep sea, and seawater [5, 7]. This probably indicates that there are very small niches of alkalinity distributed in ordinary environments. It is also considered that the evolutionary adaptation is easier from neutralophiles to alkaliphiles than from mesophiles to thermophiles. It is expected that whole enzyme systems should change from mesophilic enzymes to thermophilic enzymes for the evolutionary change from mesophiles to thermophiles.

Alkaliphilic* Bacillus* species have been isolated and studied for their environmental adaptation as typical alkaliphiles [5, 7]. The physiology of alkaline adaptation has been extensively studied on alkaliphilic species [4, 6, 8, 9]. Firstly, one of the most important strategies for alkaline adaptation is protection from alkalinity outside of their cells. It has been reported that cells of* Bacillus halodurans* C-125 produce acidic substances, such as teichuronopeptide and teichuronic acid, on their surface [10–12]. On the other hand, cells of* Bacillus pseudofirmus* OF4 produce the cell surface protein SlpA and polyglutamic acid on their surface [13]. These are acidic substances that attract H^+^ and repel OH^−^ in the extracellular environment. Protons (H^+^) are usually used in solute transport systems and torque for flagella rotation. Alkaliphilic* Bacillus* species adopt strategies for saving H^+^ usage for solute transport systems and torque for flagella rotation. They employ sodium ion solute uptake and the flagella rotation system. The above-mentioned alkaline avoidance barrier and H^+^ usage system are not well investigated in Gram-negative alkaliphiles.

In addition to the avoidance of a harmful extracellular environment and lack of the necessary ions for solute transport and flagella rotation, bioenergetics is one of the most fundamental issues for survival in alkaline environments [4, 6, 9, 14, 15]. It is considered that H^+^-deficient conditions have an extremely negative impact on energy production in microorganisms because F_1_F_0_-ATP synthase is driven by H^+^ as the torque. According to Peter Michell's chemiosmotic theory, the H^+^ motive force (Δ*p*) for driving F_1_F_0_-ATP synthase consists of a transmembrane pH gradient (ΔpH) (intracellular pH* minus* extracellular pH) and a membrane electrical potential (Δ*ψ*) (−, inside; +, outside) [16]. From the calculation of medium pH as the outer membrane pH, ATP production is impossible theoretically. It is predicted that alkaliphiles possesses mechanisms that compensate for the negative factor, that is, H^+^-deficient condition, for energy production.

Although, numbers of reported Gram-negative alkaliphiles are less than those of Gram-positive alkaliphiles, both types of alkaliphiles are distributed in soda lakes [7]. Therefore, it is very important to consider the bioenergetic problems in Gram-negative alkaliphiles to elucidate the possible environmental adaptation mechanisms that might have led to these alkaliphiles to possess a different type of cell surface membrane structure. It can be recognized that the lack of transmembrane pH gradient makes energy production difficult, for not only the Gram-positive, but also the Gram-negative bacteria. Although there are many reports on the environmental adaptation of the Gram-positive bacteria, those of the Gram negative bacteria are very few. In addition, several reviews on the bioenergetics of alkaliphilic* Bacillus* species have been published [4, 6, 9, 14, 15]. These include respiratory components and bioenergetics parameters and the relationship among them. However, there has been no review paper on bioenergetics in Gram-negative alkaliphilic bacteria. Gram-negative bacteria possess an outer membrane and a periplasmic space. These structures do not exist in Gram-positive bacteria. The periplasmic space is between the cytoplasmic membrane and the outer membrane. It has been reported that the periplasmic space comprises 40% of the total volume of the cells and it includes solutes and proteins that are involved in a wide variety of functions, ranging between nutrient binding, transport, electron transport, and alteration of substances toxic to the cell [17]. Therefore, it is expected that the periplasmic space plays an important role not only in the protection against an extracellular alkaline environments but also in retaining key proteins for bioenergetics adaptation to alkaline environments. In this review, we discuss the characteristics and physiological functions of soluble cytochromes* c* in the periplasmic space for adaptation to alkaline environments. Furthermore, we also discuss the differences between Gram-negative and Gram-positive alkaliphiles from the bioenergetics viewpoints.

## 2. Alkaliphilic* Pseudomonas*

**Figure 1:**
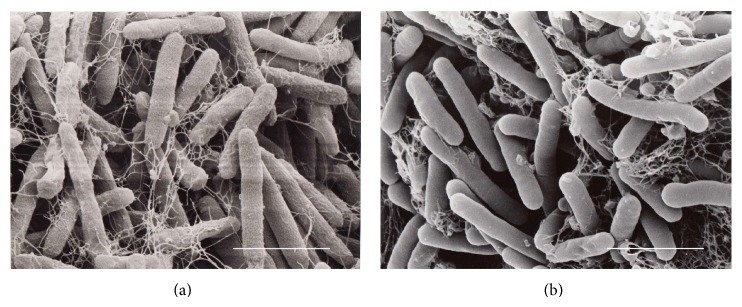
Scanning electron microscopy image of cells of platinum/palladium-coated* P*.* alcaliphila* AL15-21^T^ grown (a) at pH 10 showing the rough surface of the cells and (b) at pH 7 showing the smooth surface of the cells. Bar, 1.80 *μ*m.

**Figure 2:**
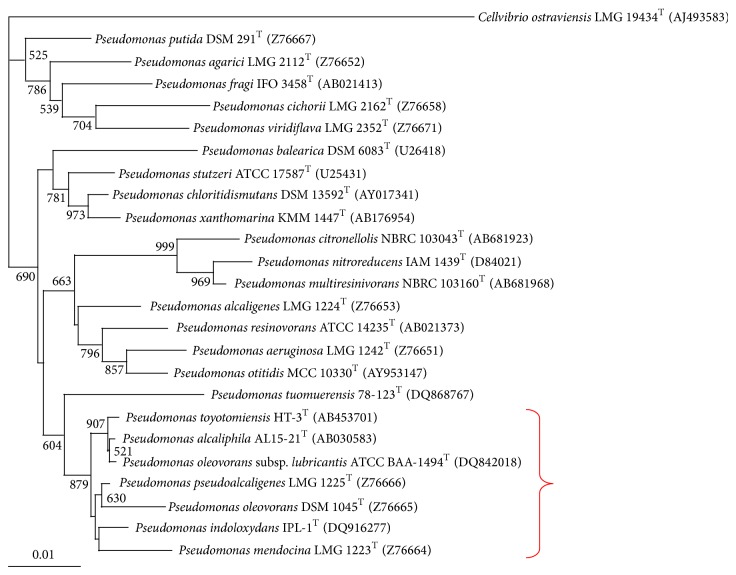
Neighbour-joining phylogenetic tree derived from 16S rRNA gene sequence data showing the position of* P. alcaliphila* AL15-21^T^ and other related* Pseudomonas* species. Numbers at branch points are bootstrap percentages based on 1000 replicates. Bar, 0.01 changes per nucleotide position. The red bracket indicates that* Pseudomonas *species can grow at pH 10.

Gram-negative alkaliphilic bacteria are less well known than Gram-positive ones. It is probably because most colonies on standard alkaline-containing media such as Horikoshi's medium are identified as* Bacillus* species when we try to isolate alkaliphilic bacteria from terrestrial environments such as soils and manure [5, 7]. However, if we try to isolate alkaliphiles from aquatic environments or particular substances such as polychlorinated biphenyl (PCB)- [18, 19] or hydrocarbon- [20] containing media, the possibility of isolating alkaliphiles other than* Bacillus* species such as* Pseudomonas* species becomes higher.* Pseudomonas* species are widely distributed in nature, such as soils, freshwater, seawater, and terrestrial and marine animals and plants [21]. Some* Pseudomonas* species are able to survive in organic solvents such as toluene [22, 23]. It is considered that* Pseudomonas* species are a metabolically and genetically diverse bacterial group. Their functions are related to the circulation of carbons and nitrogen on Earth by decomposing various organic substances and reducing nitrogen compounds. On the other hand, some of the* Pseudomonas* species are pathogenic to humans, plants, and fish. Considering their environmental distribution and metabolic and genetic diversity, it is no wonder that numerous alkaliphilic* Pseudomonas* species exist in various taxonomic groups. It has been known that* Pseudomonas pseudoalcaligenes* can grow on media with high pH [18, 19]. We have isolated* Pseudomonas alcaliphila* AL15-21^T^ from seawater obtained from the coast of Rumoi, Hokkaido, Japan, by using a general medium for the isolation of alkaliphiles, which contains peptone, yeast extract, and a metal mixture [24]. A scanning electron microscopy image of the cells of platinum/palladium-coated* P*.* alcaliphila* AL15-21^T^ grown at pH 10 shows a rough surface, whereas the cells grown at pH 7 show a smooth surface (Figure 1). We have also isolated* Pseudomonas toyotomiensis* from a surface soil immersed in hydrocarbon-containing hot spring water using a medium containing hydrocarbon as the sole carbon source [20].* P*.* pseudoalcaligenes*,* P*.* alcaliphila,* and* P*.* toyotomiensis* are on the same node in the phylogenetic tree based on the 16S rRNA gene sequence (Figure 2).* Pseudomonas mendocina* and* Pseudomonas oleovorans* are also located on the same node with these alkaliphilic* Pseudomonas* species and as far as we have examined, they also were able to grow at high pH (pH 10). Therefore, it is considered that this phylogenetic group belongs to alkaliphiles. Nine strains of alkaliphiles belonging to the genus* Pseudomonas* were isolated from a Hungarian soda lake [25]. They are closely related to* P*.* alcaliphila* and* P*.* pseudoalcaligenes *with 16S rRNA gene sequence similarities higher than 98%. Recently, a novel alkaliphilic* Pseudomonas* species, “*Pseudomonas yangmingensis,*” has been isolated from a sample of soil mixed with water from a hot sulfur spring in Taiwan [26]. This strain does not belong to the node that includes* P*.* alcaliphila*. This suggests that the possibility that undiscovered alkaliphilic* Pseudomonas* species still exist in nature.

## 3. Bioenergetic Background

### 3.1. Chemiosmotic Theory and Growth

There is less information on alkaliphilic* Pseudomonas* species than on alkaliphilic* Bacillus* species. First, we would like to describe the elucidated bioenergetics mechanisms in alkaliphilic* Bacillus* species as background references on* Pseudomonas *species. The effect of growth pH on the growth characteristics and bioenergetics parameters has been studied in the steady state under pH-controlled culture at various pHs. Generation times of 54 and 38 min were obtained at medium pHs controlled at 7.5 and 10.6, respectively, in the facultatively alkaliphilic bacterium* Bacillus pseudofirmus* OF4 [27]. The obligate alkaliphilic bacterium* Bacillus clarkii* strain K24-IU exhibits a higher growth rate (*μ*_max⁡_ = 0.33 h^−1^) than the neutralophilic bacterium* Bacillus subtilis* IAM 1026 (*μ*_max⁡_ = 0.26 h^−1^) in batch culture [28]. These growth characteristics of alkaliphilic* Bacillus* species contradict the deficiency of bioenergetics parameter owing to the reversed transmembrane pH gradient in alkaliphilic* Bacillus *in alkaline conditions.

It is considered that F_1_F_0_-ATP synthase produces ATP energized by Δ*p* across the cell membrane [16]. According to the formula in Peter Mitchell's chemiosmotic theory, Δ*p* production derived based on the Gibbs free energy can be calculated as

(1) $$\begin{array}{c} \Delta p=\Delta \psi -Z\Delta \text{pH}\;\;Z=\frac{2.3RT}{F}=59, \end{array}$$

where Δ*ψ* is the electrical potential across the membrane (−, inside; +, outside) and ΔpH is the transmembrane pH gradient (intracellular pH* minus* extracellular pH). The value *Z* = 59 can be calculated as 2.3 (coefficient for exchange from natural legalism to common legalism) × gas constant (*R* = 8.315 J/mol) × absolute temperature (298 K = 25°C)/Faraday constant (*F* = 96.485 kj/[v·mol]). Although the Δ*ψ* values of alkaliphilic* Bacillus* species are relatively higher than those of neutralophilic bacteria, they cannot compensate for the reversed ΔpH for ATP production theoretically if the medium pH is regarded as the extracellular pH [4, 6, 15]. Therefore, if we consider the medium pH as the extracellular pH, it is impossible to produce ATP by the theoretically calculated Δ*p*. Therefore, it is expected that the pH of the outer surface of the membrane is lower than the medium pH. The evidence proved that the same alkaliphilic* Bacillus* species exhibit a higher growth rate and extent even during growth at pH 10, as described above [29].

### 3.2. Proton Behavior at Outer Surface of Membrane under Neutralophilic Condition

It seems that the H^+^ transfer along the outer surface of the membrane under high pH is difficult owing to the predicted diffusion of H^+^ attracted by the force from the H^+^-deficient bulk environment. In the neutralophilic environment, the rapid movement of translocated H^+^ by the respiratory chain or bacteriorhodopsin to the channel gate of F_1_F_0_-ATP synthase on the outer surface of the membrane has been reported. In most experiments, the rapid transfer of H^+^ on the outer surface of the cellular membrane has been demonstrated using a fluoresceine indicator particularly attached to the cysteine residue in the native protein [30, 31]. The H^+^ transfer on the outer surface of the membrane was enhanced by the existence of a proton-collecting antenna consisting of carboxylate and histidine residues [32, 33]. On the other hand, in a report, the H^+^ attraction at the outer surface of the membrane is accounted for by the H^+^-attracting force owing to the negative charge of a membrane phospholipid [34]. As reported previously, even in the neutralophilic condition, H^+^ transfer along the outer surface of the membrane is clearly a fundamental mechanism for the effective production of energy. Therefore, it must not be true that the pH at the outer surface of the membrane is the same as that of the medium. It has been reported that alkaliphilic* Bacillus* species generate a larger transmembrane electrical gradient (Δ*ψ* = −180 ~ −210 mV) [4, 6, 15] than the neutralophilic* Bacillus subtilis* (Δ*ψ* = −122 ~ −130 mV) [35, 36]. Although it has been considered that Δ*p* calculated from the medium pH (Δ*p* = ca.−40) is not sufficient for ATP production, the large Δ*ψ* may play a role in compensating for the adverse H^+^ concentration in the environment.

### 3.3. Behavior of Translocated H^+^ in Alkaliphilic* Bacillus*

Peter Mitchell (1968) advocated the H^+^ well concept that accounts for the bioenergetics equivalence of the chemical (ΔpH) and electrical (Δ*ψ*) components of the H^+^ motive force (Δ*p*). The H^+^ well was defined as an H^+^-conducting crevice passing down into the membrane dielectric and able to accumulate H^+^ in response to the generation of either Δ*ψ* or ΔpH. The H^+^ well concept implies that an H^+^ in such a well would sense both the surface pH changes and the changes in Δ*ψ*. The outer surface of the membrane senses the changes in Δ*ψ* and/or in surface pH and would drive H^+^ either inside or outside the well. Mulkidjanian advocated that the H^+^ well is constructed on the basis of the desolvation penalty for transferring H^+^ into the membrane core [37]. Retardation of the H^+^ translocated by the respiratory chain on the outer surface of the membrane was demonstrated by* B*.* clarkii *K24-1U grown at pH 10. In addition, the retardation of H^+^ at the outer surface of membrane was disrupted by valinomycin. This indicates that there is possibility that an H^+^ trap occurred because H^+^ on the outer surface of the membrane senses the presence of Δ*ψ* [29].

## 4. Electron Transfer Coupled to H^+^ Transfer in Cytochrome* c*

It has been considered that cytochrome* c* is solely an electron transfer protein. H^+^-coupled electron transfer in cytochrome* c* has been demonstrated by Murgida and Hildebrandt [38]. The electron transfer dynamics of horse heart cytochrome* c* of self-assembled monolayers (SAM) adsorbed on an Ag electrode formed by different *ω*-carboxyl alkanethiols of different chain lengths (C_2_–C_16_) was monitored by changing the electrode potential. The electron transfer rate between the cytochrome* c* and the Ag electrode was slower when D_2_O (33 s^−1^) rather than H_2_O (132 s^−1^) and was used as the aqueous solution when the *ω*-carboxyl alkanethiol chain length was short (C_2_; distance between the cytochrome* c* and the electrode: 6.3 Å).On the other hand, there is no difference in the electron transfer rate (0.073–0.074 s^−1^) regardless of whether D_2_O or H_2_O was used as the aqueous solution when the *ω*-carboxyl alkanethiol chain length was long (C_16_: 24 Å). It is considered that the H^+^-exchange-coupled electron transfer may be a rate-limiting step only if the velocity of electron transfer between the cytochrome* c* and the Ag electrode is high. This phenomenon has not been observed for cytochrome* c* in solution. Therefore, it is considered that the H^+^ transfer rate between the cytochrome* c* and the aqueous solution is affected by the change in the electric field when the electron was taken in and out during the electron-transfer-coupled H^+^ transfer. This suggested that the H^+^ transfer rate between the cytochrome* c* and the aqueous solution is affected by the change in the transmembrane potential (Δ*ψ* and/or ΔpH) during the electron-transfer-coupled H^+^ transfer in biological membranes.

## 5. Bioenergetics and Function of the Cytochrome* c* in Alkaliphilic* Bacillus*

Alkaliphilic* Bacillus* spp. have several strategies for cutting corners of H^+^ usage in several functions such as solute transport and torque of flagella rotation by using Na^+^ [6]. However, they use H^+^ as the torque of F_1_F_0_-ATP synthase rotation. Therefore, they may have several strategies in order not to waste the precious H^+^ across the membrane. The H^+^ transfer or recycle event will happen in the vicinity of membrane as mentioned above. Cytochrome* c* may occur at an important function for the efficient use of H^+^ at the vicinity of outer surface membrane. On the other hand, alkaliphilic* Bacillus* spp. possess larger Δ*ψ*. This suggests that Δ*ψ* has important functions including the compensation of deficient ΔpH. The presence of cytochrome* c* has been considered important in alkaliphilic* Bacillus* species for adaptation to alkaline environments. Mutants of obligate alkaliphiles, which cannot grow under alkaline condition, exhibit a much lower cytochrome* c* content than the wild type strain [39]. Facultative alkaliphiles, such as* Bacillus pseudofirmus* OF4 and* Bacillus cohnii* YN-2000 exhibit a higher cytochrome* c* content when they are grown at alkaline pH than when they are grown at neutral pH [40, 41]. However, the induction of cytochrome* c* is not always observed in all alkaliphilic* Bacillus *species grown at high pH [42]. Membrane-anchor-less, low-molecular-mass cytochromes* c* have been isolated from alkaliphilic* Bacillus* species (including* Sporosarcina pasteurii*) and characterized [41, 43–45]. All these cytochromes* c* were determined to have low redox potential (+47~+95 mV) compared with those from neutralophiles (+178 mV in* Bacillus subtilis*) [46] by redox titration. The low redox potential of cytochromes* c* produces a large redox potential difference between its electron accepter, cytochrome* a* in the terminal oxidase. This large redox potential may play an important role in electron transfer between cytochrome* c* and cytochrome* c* oxidase at high Δ*ψ* across the cellular membrane. These cytochromes* c* from alkaliphiles exhibit acidic nature (p*I* = 4.0–4.2) compared with those from neutralophilic* Bacillus* species (p*I* = 5.2–9.7) [4]. It has been reported that the outer segments of several membrane proteins exhibit extensively reduced amounts of basic amino acids and increased amounts of acidic amino acids. Membrane-bound cytochromes* c* in Gram-positive bacteria also exist at the outer surface of membrane. The acidic nature of these proteins will result in a negative charge to attract H^+^ and expel OH^−^ at the outer surface of the cellular membrane.

Membrane-bound cytochromes* c* are considered to bind to the cellular membrane via a diacylglyceryl-cysteine moiety. Although several diacylglyceryl-cysteine moieties attached to cytochromes* c* have been isolated from several Gram-positive bacteria, there has been no report on the diacylglyceryl-cysteine moiety attached to cytochromes* c *from alkaliphilic* Bacillus*. Ogami et al. purified and characterized a cytochrome* c* with a diacylglyceryl-cysteine moiety attached, namely, cytochrome* c*-550, from an obligate alkaliphilic* Bacillus clarkii *K24-1U [28]. Cytochrome* c*-550 binds to fatty acids of C_15_, C_16_, and C_17_ chain lengths via glycerol-Cys^18^. The cytochrome* c* is an acidic protein (predicted p*I* = 4.1 based on amino acid composition). It contains only two basic amino acids including histidine of the heme* c* axial ligand. This cytochrome* c* contains a conspicuous amino acid sequence of Gly^22^–Asn^34^, in comparison with the cytochromes* c* of other neutralophilic or facultative alkaliphilic* Bacillus* species. Similar amino acid sequences have been observed only for obligate alkaliphiles. The amino acid sequence of Gly^22^–Asn^34^ location corresponds to the intermediate location between N-terminal and first *α*-helical structure from the N-terminal. It can be predicted that the sequence locates the surface of the cytochrome* c* molecule. The amino acid sequences are rich in asparagine (Asn) residue. An Asn residue located in H^+^ transfer pass way has been reported in heme-copper oxidase [47]. The conspicuous amino acid sequence may have an important function in retaining or transferring H^+^ from the outer surface of the membrane under alkaline pH condition by its contribution for the formation of H-bound network on the outer surface of the membrane. The midpoint redox potential (*E*_*m*,7_) of cytochrome* c*-550 as determined by redox titration was +83 mV. On the other hand, the midpoint redox potential (*E*°′) value at pH 7 determined by cyclic voltammetric measurement was +7 mV [28]. It is considered that the redox potential of cytochrome* c*-550 becomes even lower under a changing electric field when electrons are taken up and out. This may be related to the midpoint redox potential change of cytochrome* c* depending on the Δ*ψ* across the cellular membrane. In addition, although it is not certain whether the ambient pH affects the midpoint redox potential of cytochrome* c*-550, there is a possibility that the ambient pH or ΔpH affects the midpoint redox potential of cytochrome* c*-550. This produces an even larger redox potential difference between cytochrome* c*-550 and the electron accepter, cytochrome* a*. The translocation of H^+^ in presence of a larger Δ*ψ* can be predicted. The cytochrome* c* faction that lowers the redox potential in the presence of a large Δ*ψ* may play an important role in translocating the positively charged H^+^ from the intracellular to extracellular side of the membrane.

It is considered that the regulation of H^+^-coupled electron transfer of cytochrome* c* plays an important role in the adaptation of alkaliphilic* Bacillus* species to alkaline environments. From the facts described above, the redox state (presence or absence of electron), amino acid sequence, acidic nature of cytochrome* c*, Δ*ψ*, ΔpH (or ambient pH), and redox potential change may affect the H^+^ behavior in H^+^-coupled electron transfer in cytochrome* c*.

Although alkaliphilic* Bacillus* species produce a large amount of cytochrome* c* during their growth at high pH, the respiratory rates are not high. This fact suggests that cytochrome* c* stores electrons rather than accelerates electron transport in the respiratory chain. In addition, the characteristics of cytochrome* c* together with electron storage in the cytochrome* c* and Δ*ψ* are related to H^+^ accumulation. It has been reported that facultative alkaliphilic* Bacillus cohnii* YN-2000 produces higher amounts of a nonproteinaceous substance at pH 10 than at pH 7 [47]. This nonproteinaceous substance plays a role as a substituent of cytochrome* c* oxidase in terminal oxidation in the respiratory chain [48]. It is considered that it does not have a function in the electron-transfer-coupled H^+^ transfer in the respiratory chain. These facts described above suggested that the respiratory chain of alkaliphilic* Bacillus* species not only has a function in the electron-coupled H^+^ transfer across the membrane but also in the electron and H^+^ storage for ATP production. Hence, there is a possibility that the cytochrome* c* accumulated in large amounts on the outer surface of membrane functions as an electron and H^+^ condenser.

## 6. Production of Soluble Cytochromes* c* and Their General Characteristics in* P*.* alcaliphila*

As described above, Gram-negative bacteria have a periplasmic space, whereas Gram-positive bacteria do not have a periplasmic space. Therefore, Gram-negative bacteria have soluble cytochrome* c*, whereas Gram-positive bacteria only have membrane-bound cytochrome* c*. The cytochrome* c* contents in the soluble fraction at pH 7–10 under low- and high-aeration intensities in Gram-negative facultative alkaliphilic* P*.* alcaliphila* AL 15-21^T^ have been estimated [49]. The cytochrome* c* content in the soluble fraction is higher in the cells grown at pH 10 than at pH 7 under low-aeration intensity. In addition, the cytochrome* c* content is higher under low-aeration intensity than under high-aeration intensity. The highest of cytochrome* c* content is observed in the cells grown at pH 10 under low-aeration intensity. The cytochrome c content in the cells grown at pH 10 under low-aeration intensity (0.47 ± 0.05 nmol·mg protein^−1^) was 3.6-fold higher than that in the cells grown at pH 7 under high-aeration intensity (0.13 ± 0.01 nmol·mg protein^−1^). In the soluble fraction of* P*.* alcaliphila* AL-15-21^T^, three types of soluble cytochrome* c*, namely, cytochrome* c*-552 [49], cytochrome* c*-554, and cytochrome* c*-551, have been found.

### 6.1. Properties of Cytochrome* c*-552

**Figure 3:**
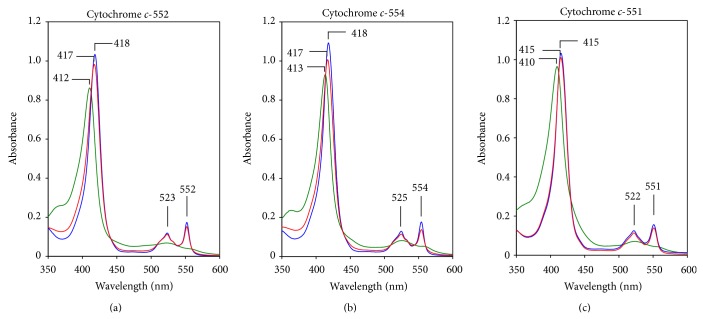
Absorption spectra of resting (red line), reduced (blue line), and oxidized (green line) cytochrome* c*-552 (a), cytochrome* c*-554 (b), and cytochrome* c*-551 (c) from* P*.* alcaliphila* AL15-21^T^ showing *α*, *β*, and Soret bands. The protein was reduced with sodium dithionite and oxidized with potassium ferricyanide.

**Figure 4:**
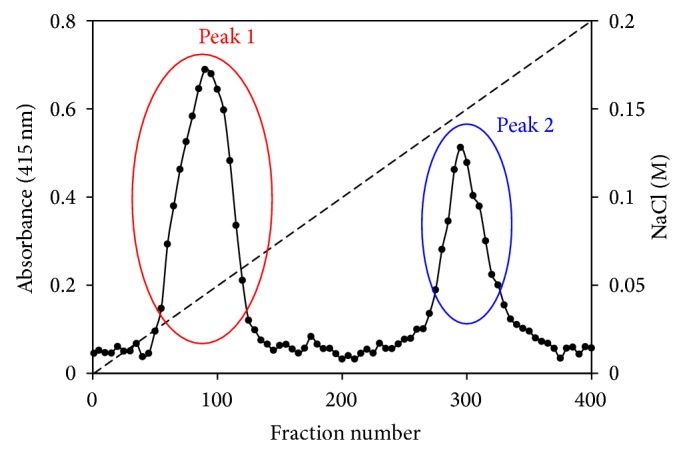
Elution profile of anion-exchange chromatography (QAE-Toyopearl) loaded on the cell extract of* P*.* alcaliphila* AL15-21^T^ grown at pH 10 under low aeration intensity. The cell extract was prepared by cell disruption using a French press. The first (peak 1) and second (peak 2) peaks indicate cytochrome* c*-552 and a mixture of cytochromes* c* other than cytochrome* c*-552 (cytochrome* c*-554 and* c*-551), respectively. The percentages relative to total cytochromes* c* of peaks 1 and 2 are 64% and 36%, respectively.

Cytochrome* c*-552 comprises 64% of the total soluble cytochrome* c* (Figure 3). The remaining cytochromes* c*,* c*-551, and* c*-552 comprise the remaining 36%. Therefore, cytochrome* c*-552 is the major soluble cytochrome* c* in* P*.* alcaliphila*. The absorption spectrum of the resting state of cytochrome* c*-552 is very similar to that of the fully reduced state (Figure 4). The *α*, *β*, and Soret peaks of the purified cytochrome* c*-552 in the resting state are at 552, 523, and 417 nm, respectively. On the other hand, the peaks of its fully reduced state are at 552 (*α*), 523 (*β*), and 418 (Soret) nm. In its fully oxidized form, the Soret peak is at 412 nm. Its molecular mass determined by sodium dodecyl sulfate- (SDS-) poly acrylamide gel electrophoresis (PAGE) is 7.5 kDa. This mass is smaller than those of typical cytochromes* c*; for example, soluble cytochromes* c* purified from* Pseudomonas aeruginosa *are reported to be 9–15 kDa. Although the p*I* of cytochrome* c*-552 predicted on the basis of its amino acid composition is 5.7, the p*I* determined by isolectric focusing is 4.3. Although its p*I* is slightly higher than those of the cytochromes* c* of alkaliphilic* Bacillus* species (p*I* = 3.4–4.0), the acidic nature of cytochrome* c*-552 is similar to those from alkaliphilic* Bacillus*. The midpoint redox potentials (*E*_*m*,7_) of cytochrome* c*-552 as determined by redox titration and cyclic voltammetry are +228 and +224 mV, respectively. Although these values are much higher than those of cytochrome* c* from alkaliphilic* Bacillus* species (+47 mV–+95 mV), they are lower than those of cytochrome* c*-550 and* c*-552 from neutralophilic* Pseudomonas aeruginosa*, that is, +280 mV and +286 mV, respectively.

**Figure 5:**
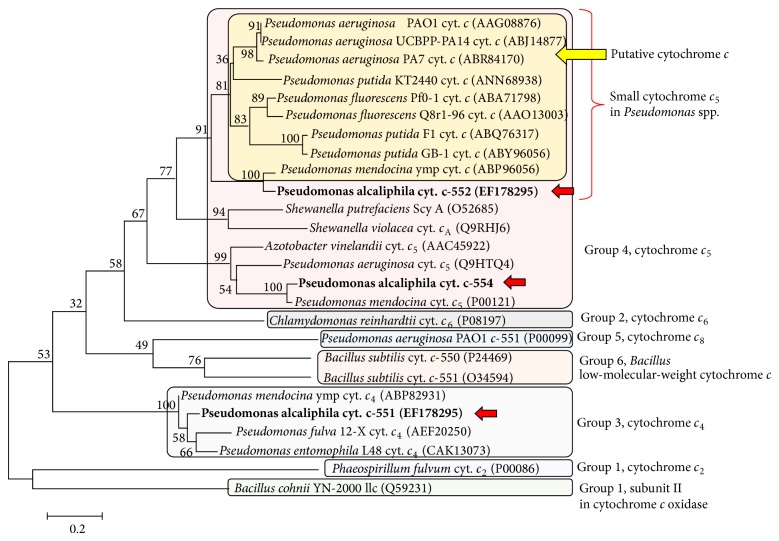
Phylogenetic tree for cytochromes* c* of* P*.* alcaliphila* AL15-21^T^ based on the amino acid sequence. These are indicated by red arrows. The phylogenetic tree was constructed by the neighbour-joining method. The numbers at nodes are bootstrap values based on 1000 replicates. Bar, 0.01 changes per nucleotide position. The red bracket indicates the small cytochrome* c*_5_ in* Pseudomonas* species. The yellow marking and blue bold arrow indicate putative small cytochrome* c*_5_ observed in the determined whole genome sequence.

The gene coding for cytochrome* c*-552 reveals that it consists of 96 amino acids with 19 residues as the signal peptide located at the N-terminal sequence. The amino acid sequence deduced from the gene sequence revealed a single heme-binding sequence, Cys-X-X-Cys-His (monoheme cytochrome* c*). The amino acid sequence of cytochrome* c*-552 exhibited a relatively high similarity with the amino acid sequence encoded in the genes of* Pseudomonas* species. These are assigned as putative cytochrome* c* from the determined full genome sequence of* Pseudomonas *species. Therefore, the proteins produced from these genes have never been purified and characterized. The amino acid sequence of cytochrome* c*-552 exhibited similarity with those of putatively assigned cytochromes* c* as follows:* Pseudomonas mendocina* putative cytochrome* c* (gene accession number in Genbank/EMBL/DDBJ:ABP82930; 91.6% identity),* Pseudomonas aeruginosa* putative cytochrome* c* (ABR84170; 69.8%), and* Pseudomonas fluorescens* putative cytochrome* c* (ABA71798; 61.5%). The high sequence similarity between cytochrome* c*-552 and cytochrome* c* of* P*.* mendocina* may reflect the phylogenetic relationship between* P*.* alcaliphila* and* P*.* mendocina*. As described above,* P*.* mendocina* can also grow at pH 10. There is a possibility that the presence of a cytochrome* c* similar to cytochrome* c*-552 plays an important role in the adaptation to alkaline environments. On the basis of phylogenetic analysis based on the amino acid sequence, cytochrome* c*-552 is classified as small cytochrome* c*_5_ belonging to group 4 in class I cytochrome* c* (Figure 5).

### 6.2. Properties of Cytochrome* c*-551

The absorption spectrum of the resting state of cytochrome* c*-551 is identical to that of the fully reduced state (Figure 4). The *α*, *β*, and Soret peaks of the purified cytochrome* c*-551 in the resting state are at 551, 522, and 415 nm, respectively. The peaks of its fully reduced state are at 551 (*α*), 522 (*β*), and 415 (Soret) nm. In its fully oxidized form the Soret peak is at 410 nm. The molecular mass determined by SDS-PAGE is 19 kDa. Its p*I* predicted on the basis of the amio acid composition is 5.4.

The gene sequence encoding cytochrome* c*-551 is located downstream of the gene sequence encoding cytochrome* c*-552. The gene coding for cytochrome* c*-551 reveals that it consists of 201 amino acids with 20 residues as the signal peptide located at N-terminal sequence. The amino acid sequence deduced from the gene sequence revealed two heme-binding sequences, Cys-X-X-Cys-His (diheme cytochrome* c*). The amino acid sequence of cytochrome* c*-551 exhibited similarity with that of* Pseudomonas mendocina* cytochrome* c*_4_ (gene accession number in Genbank/EMBL/DDBJ:ABP82931; 98.0% identity). On the basis of phylogenetic analysis based on the amino acid sequence, cytochrome* c*-551 is classified as cytochrome* c*_4_ belonging to group 3 in class I cytochrome* c* (Figure 5).

### 6.3. Properties of Cytochrome* c*-554

The absorption spectrum of the resting state of cytochrome* c*-554 is very similar to that of the fully reduced state (Figure 4). The *α*, *β*, and Soret peaks of the purified cytochrome* c*-554 in the resting state are at 554, 525, and 418 nm, respectively. The peaks of its fully reduced state are at 554 (*α*), 525 (*β*), and 417 (Soret) nm. In its fully oxidized form, the Soret peak is at 413 nm. Its molecular mass determined by SDS-PAGE is 11 kDa. Its p*I* predicted on the basis of the amino acid composition is 5.5.

The gene coding for cytochrome* c*-554 reveals that it consists of 137 amino acids with 21 residues as the signal peptide located at N-terminal sequence. The deduced amino acid sequence from the gene sequence revealed a single heme-binding sequence, Cys-X-X-Cys-His (monoheme cytochrome* c*). The amino acid sequence of cytochrome* c*-554 exhibited similarity with those of* P*.* mendocina* cytochrome* c*_5_ (gene accession number in Genbank/EMBL/DDBJ:P00121; 93.1% identity) and* P*.* aeruginosa* cytochrome* c*_5_ (gene accession number in Genbank/EMBL/DDBJ: Q9HTQ4; 70.6% identity). On the basis of phylogenetic analysis based on the amino acid sequence, cytochrome* c*-554 is classified as cytochrome* c*_5_ belonging to group 4 in class I cytochrome* c* (Figure 5).

## 7. Redox Properties of Cytochrome* c*-552

Cytochrome* c*-552, a small cytochrome* c*_5_ belonging to group 4 in class I cytochrome* c*, has been firstly purified and characterized. However, further characterization and clarification of the physiological function of cytochrome* c*-552 is difficult because purification of a large amount of the protein is difficult given that the amount of expressed protein from* P*.* alcaliphila* is very small in comparison with the amount of concomitantly existing background proteins. Therefore, if purification is started from a large amount of cells, the first purification step is very difficult to manage owing to the large amount of protein. To obtain a large amount of purified cytochrome* c*-552, a recombinant cytochrome* c*-552 expression system was constructed by the transformation of the plasmid (pKK223-3[c552]) carrying the cytochrome* c*-552 gene and the plasmid (pEC86) carrying the gene cluster of* ccmA-H*, requiring the assembly of a* c*-type cytochrome in* Escherichia coli* under anaerobic growth condition. The yield of cytochrome* c*-552 from recombinant* E*.* coli* is 2.8 mg·L^−1^ of culture medium, which is 76.4-fold larger than that from native cells. Analytical data such as absorption spectra,* E*_*m*,7_, molecular mass determined by SDS-PAGE and MALDI-TOF MS and N-terminal amino acid sequence exhibited that the recombinant protein is exactly the same as the native cytochrome* c*-552.

Changes in the redox potential (*E*°′) depending on the pH were estimated using the recombinant cytochrome* c*-552. The* E*°′ values at pHs, 6, 7, and 8 are 223, 224, and 226 mV, respectively (average: 224 mV). On the other hand, the* E*°′ values at pHs 9 and 10 are 217 and 218 mV, respectively (average: 218 mV). Although these determined values are similar to one another, the* E*°′ values are slightly decreased at pHs 9 and 10 in comparison to those of pHs 6–8. This small change may contribute electron retaining ability of cytochrome* c*-552 at pHs 9-10. The* E*_*m*,7_ of cytochrome* c*-552 is lower than that of cytochrome* c*-550 of neutralophilic* Pseudomonas aeruginosa*. Although the high affinity of electrons in cytochrome* c*-552 cannot be accounted for only by the* E*°′ values, the lower* E*°′ value of cytochrome* c*-552 than that of the neutralophile may have a meaning in adjusting the electron transfer between cytochrome* c*-552 and its electron acceptor, cytochrome* c* oxidase, in the adapting to physiological conditions.

The reduction rate of cytochrome* c*-552 was estimated using native cytochrome* c*-552 by estimating the reduction rate under anaerobic condition (pHs 8.5 [100 mM Tris-HCl buffer] and 10 [100 mM glycine-NaOH buffer] and the presence or absence of 10 *μ*M TMPD) [49]. Cytochrome* c*-552 was almost fully reduced after 40 h of incubation at pH 8.5, while it was fully reduced after 4 h at pH 10 in the presence of TMPD. The reduction rate exhibited a first-order reaction with reaction constants of 0.07 and 0.56 h^−1^ at pHs 8.5 and 10, respectively. The reduction of cytochrome* c*-552 occurs at pH 8.5 only in the presence of TMPD, while it is observed at pH 10 even in the absence of TMPD. Cytochrome* c*-552 is almost fully reduced at pH 10 in the absence of TMPD with a reaction constant of 0.04 h^−1^. These results indicated that the electron mediator, TMPD, accelerates the reduction of cytochrome* c*-552 and glycine reduces cytochrome* c* at pH 10.

The oxidation rate of cytochrome* c*-552 at pHs 6–10 is estimated using 100 mM buffers as follows: phosphate-NaOH buffer for pHs 6 and 7, Tris-HCl buffer for pH 8, and sodium carbonate buffer for pHs 9 and 10 [50]. Cytochrome* c*-552 oxidized very slowly at pHs 8–10 with the slowest at pH 8, while it oxidized rapidly at pHs 6-7. On the other hand, the oxidation rates of horse heart cytochrome* c* are high at pHs 6–10. The results described above indicate that cytochrome* c*-552 possesses the distinctive ability to retain its reduced state at pHs 8–10. As described above, the electron-transfer-coupled H^+^ transfer in cytochrome* c* has been reported. The electron-transfer-coupled H^+^ transfer is possible if cytochrome* c*-552 retention of H^+^ on the basis of the negative charge of electron in its reduced state. A high concentration of cytochrome* c*-552 in the periplasmic space will contribute to the retention of H^+^ in the extracellular membrane space.

## 8. Role of Cytochrome* c*-552 in Respiratory System

For the search of an electron acceptor of cytochrome* c*-552, cytochrome* c* oxidase was purified from* P*.* alcaliphila*. The cytochrome* c* oxidase obtained from the solubilized membrane of* P*.* alcaliphila* is a* cb*-type cytochrome* c* oxidase, which shows an absorption Soret peak at 412 nm in the oxidized state. In the reduced state, it shows a Soret peak at 426 nm with a distinct shoulder at 418 nm. In the visible region, peaks at 558, 553, 529, and 523 nm are observed in the reduced form. The peaks at 418 (shoulder), 523 and 553 nm are characteristic of ferrous* c*-type hemes. On the other hand, the peaks at 426, 529 and 558 nm are characteristic of ferrous* b*-type hemes. The* cb*-type cytochrome* c* oxidase shows a higher absorption intensity for* b*-type hemes than for* c*-type hemes, whereas* Pseudomonas stutzeri *cytochrome* cbb*_3_ oxidase shows a higher absorption intensity for* c*-type hemes [51]. Actually* Pseudomonas stutzeri *cytochrome* cbb*_3_ oxidase contains two* b*-type and three* c*-type hemes. The* cb*-type cytochrome* c* oxidase exhibits bands showing molecular masses of 55, 42 and 32 kDa in SDS-PAGE.* Pseudomonas stutzeri *cytochrome* cbb*_3_ oxidase exhibits bands showing molecular masses of 41, 35 and 26 kDa [52], while the molecular masses predicted from the gene sequence are 53, 197, 34,984 and 23,463 Da. These molecular masses are very similar to those of the cytochrome* cbb*_3_ oxidase from* P*.* mendocina*. The molecular masses of subunits of the cytochrome* cbb*_3_ oxidase from* P*.* mendocina* predicted from the gene sequence are 53, 34, 22 and 6 kDa (CP000680-2580-2583). The cytochrome* c* oxidase activity of the* cb*-type cytochrome* c* oxidase was measured spectrophotometrically using recombinant* P*.* alcaliphila* cytochrome* c*-552 and horse heart cytochrome* c* as the electron donors. The cytochrome* c* oxidase activities were 9.6 and 8.2 *μ*mol·min^−1^·mg^−1^ when* P*.* alcaliphila* cytochrome* c*-552 and horse heart cytochrome* c* were used as the electron donors, respectively.

## 9. Contribution of Cytochrome* c*-552 for Alkaline Adaptation

**Figure 6:**
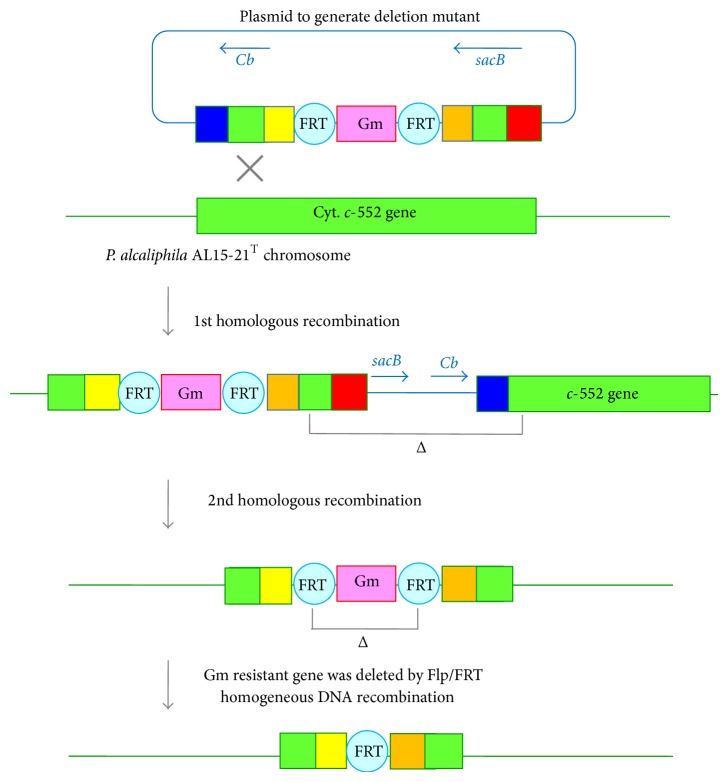
Scheme of producing unmarked cytochrome* c*-552-deficient mutant of* P*.* alcaliphila* AL15-21^T^ by homologous recombination. The mutant fragment generated by overlap extension PCR is cloned into the pDONR221 and then recombined into the pEX18ApGW plasmid by gateway-recombinational cloning. The resulting suicide vector is transferred to* P*.* alcaliphila* AL15-21^T^. The plasmid-borne deletion mutation is exchanged with the corresponding part in the chromosome. The merodiploid state is resolved by* sacB*-mediated sucrose counterselection in the presence of gentamycin. Finally, the gentamycin resistance marker (Gm^R^) is deleted by Flp/FRF homologues DNA recombination.* sacB*:* Bacillus subtilis* levansucrase-encoding gene,* Cd*: carbenicillin resistance marker.

To understand the physiological function of cytochrome* c*-552 in* P*.* alcaliphila* AL15-21^T^ [53], an antibiotic-marker-less cytochrome* c*-552-deficient mutant has been constructed following the method of Choi and Schweizer [54]. A mutant fragment containing the 5′ and 3′ flanking regions of the target gene, that is, chromosomal cytochrome* c*-552 gene, plus an antibiotic resistance maker gene, Gm^R^, originated from the pPS856 plasmid is contracted by a PCR overlap extension. The resulting mutant fragment was cloned into the Gateway vector pDONR 221 by BP clonase reaction and then recombined into the Gateway-compatible gene replacement suicide vector pEX18ApGW by LR clonase reaction (Figure 6). The plasmid-borne deletion allele is then transformed into* P*.* alcaliphila* AL15-21^T^ by electroporation and recombined into the bacterial chromosome. The mutant fragment is integrated into the target gene by a single- or double-crossover event. Merodiploids via the integration of the suicide plasmid by a single-crossover event are resolved by sucrose selection in the presence of the gentamycin for deletion of the wild type gene. Finally, the antibiotic resistance marker gene (Gm^R^) is subsequently deleted from the chromosome by Flp recombination reaction.

**Figure 7:**
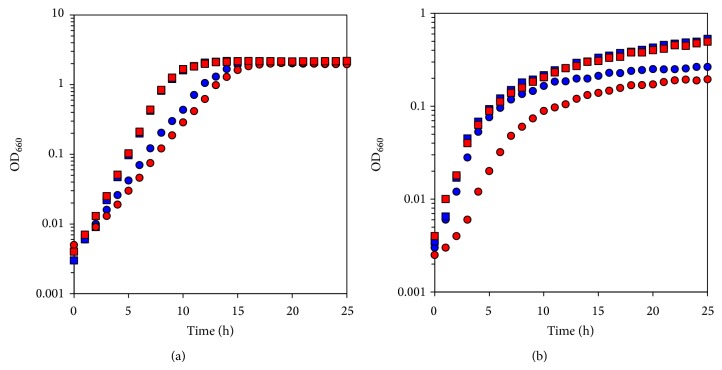
Time course of growth of* P*.* alcaliphila* AL15-21^T^ (wild type; blue symbols) and cytochrome* c*-552 deletion mutant derived from wild type (mutant; red symbols) grown under (a) high- and (b) low-aeration intensities at pH 7 (squares) and pH 10 (circles). The reproducibility of the results was ascertained by performing three independent experiments.

The growth characteristics of wild type* P*.* alcaliphila* AL15-21^T^ and the cytochrome* c*-552 deletion mutant were examined at pHs 10 and 7 under low- and high-aeration intensities (Figure 7). The maximum specific growth rate (*μ*_max⁡_ [h^−1^]) and maximum cell turbidity (OD_660,max⁡_) of the two strains were estimated. Although the growth under the low-aeration intensity is inferior to that under the high-aeration intensity, initial growth rate is higher under the low-aeration intensity. This is probably related to the high oxygen tension that inhibits the growth initiation. Significant differences in growth parameters concomitant with differences in the growth curve are observed at pH 10 under the low-aeration intensity between wild type* P*.* alcaliphila* AL15-21^T^ (*μ*_max⁡_ [h^−1^] = 0.911, OD_660,max⁡_ = 0.27) and the cytochrome* c*-552 deletion mutant (*μ*_max⁡_ [h^−1^] = 0.709, OD_660,max⁡_ = 0.21). The *μ*_max⁡_ (h^−1^) and OD_660,max⁡_ of the deletion mutant decreased by 22% and 25%, respectively, relative to those of the wild type. It is considered that this large difference is attributed to the large difference in the expression level of cytochrome* c*. A slight decrease was observed in the mutant in comparison with the wild type under high-aeration intensity in the *μ*_max⁡_ (h^−1^) during growth at pH 10. The *μ*_max⁡_ (h^−1^) is 0.475 and OD_660,max⁡_ is 2.06 in the wild type, whereas the *μ*_max⁡_ (h^−1^) is 0.416 (12% decrease) and OD_660,max⁡_ is 2.06 in the mutant. No significant difference in the *μ*_max⁡_ (h^−1^) between the two strains was observed during growth at pH 7 regardless of the aeration intensity. The *μ*_max⁡_ (h^−1^) is 1.063 and OD_660,max⁡_ is 0.53 in the wild type, whereas the *μ*_max⁡_ (h^−1^) is 1.063 and OD_660,max⁡_ is 0.50 in the mutant during growth under low-aeration intensity. The *μ*_max⁡_ (h^−1^) is 0.693 and OD_660,max⁡_ is 2.18 in the wild type, whereas the *μ*_max⁡_ (h^−1^) is 0.693 and OD_660,max⁡_ is 2.17 in the mutant grown under high aeration intensity. Under these conditions, cytochrome* c*-552 can be substituted by other soluble cytochromes* c*, such as cytochrome* c*-551 and cytochrome* c*-554 for the function of electron transfer to the terminal oxidase, cytochrome* cb*. As described above, the largest amount of soluble cytochrome* c *production was observed at pH 10 under low-aeration intensity among the pH range of 7–10 under low- and high-aeration intensities. The difference in *μ*_max⁡_.

(h^−1^) and OD_660,max⁡_ can be attributed to the large difference in the expression level of cytochrome* c*. In other words, the contribution of cytochrome* c*-552 becomes high if the amount of total soluble cytochrome* c *is enhanced at pH 10 under low-aeration intensity in the wild type.

The oxygen consumption rate of the* P*.* alcaliphila* AL15-21^T^ cell suspension was determined. No significant difference in the growth between the wild type and the cytochrome* c*-552 deletion mutant was observed at pH 7. The oxygen consumption rates of the mutant showed a 1.4% increase and a 3.1% decrease relative to those of the wild type. On the other hand, a significant promotion of the oxygen consumption rate between the two strains grown at pH 10 was observed, regardless of aeration intensity. The oxygen consumption rate is 884 ± 27 natom O·min^−1^·mg protein^−1^ in the mutant, whereas it is 790 ± 16 natom O·min^−1^·mg protein^−1^ in the wild type under low-aeration intensity. It indicates a 12% increase in the mutant relative to that in the wild type. Under the high-aeration intensity at pH 10, the rate is 1106 ± 69 natom O·min^−1^·mg protein^−1^ in the mutant, whereas it is 948 ± 53 natom O·min^−1^·mg protein^−1^ (a 17% increase) in the wild type. These results suggest that cytochrome* c*-552 acts as an electron reserve rather than a promoter of electron transfer in the respiratory chain. This is in accordance with the characteristics of cytochrome* c*-552 that exhibits high electron- retaining ability at high pH.

The generation of H^+^ motive force by the respiratory chain may be difficult under alkaline pH (pH 10) and/or under low-aeration intensity because it denotes a transport substrate on H^+^-less and/or a terminal electron acceptor on O_2_-less condition. Cytochrome* c*-552 plays a role in retaining electrons concomitant with H^+^ by the electric charge of electrons and characteristics of the protein, that is, p*K*_*a*_ of the surface amino acid sequence of the protein. Thus, the accumulation of cytochrome* c*-552 in the periplasmic space results in the storage of electrons and H^+^ in the periplasmic space in the bacterium. We hypothesize that cytochrome* c*-552 in the periplasmic space plays the role of “condenser” of H^+^ and/or electrons for the respiratory system. This system may work given the pool of H^+^ and/or electrons using the vast periplasmic space and may partially link ordinary electron-flow-dependent H^+^ transfer across the membrane. It is considered that the system using cytochrome* c*-552 in periplasmic space has an advantage for the functioning of the respiratory system under high-pH and/or low-aeration intensity.

## 10. Conclusions and Perspective

As mentioned above, developments of a barrier such as an acidic substance, teichuronopeptide, teichuronic acid and acidic amino acid chains, around the cell surface to avoid alkaline pH are observed in alkaliphilic* Bacillus*. Although the composition of the cell barrier in* P*.* alcaliphila* is not well understood, the components and specific structure can be observed by scanning electron microscopy (Figure 1). The cells of strain grown at pH 10 exhibit a rough surface of the cells, whereas cells grown at pH 7 exhibit smooth surface. Therefore, it is considered that protection of the interface from the outer environment is fundamentally very important in many alkaliphilic bacteria, whether Gram-negative or Gram-positive bacteria.

Numerous of alkaliphilic* Bacillus* species have been known as authorized species. Although alkaliphilic* Bacillus* species exhibit diverse ecological distributions and diverse phylogenetic positions, they exhibit a distinctive phylogenetic position among* Bacillus* species. Although not so many alkaliphilic* Pseudomonas* species are authorized, the consistency of the distinctive position of alkaliphilic* Pseudomonas *species is also observed. These genera include numerous species and exhibit a wide range of distribution and exquisite adaptability to various environments in nature. These results indicate that there is a relationship between the evolutionary process and the gain in adaptability to alkaline environments.

From the bioenergetics viewpoint, transmembrane Δ*ψ*, which contributes to the H^+^ attraction at the outer surface of the cellular membrane and sustains effective energy production plays one of the most important roles in alkaline adaptation in alkaliphilic* Bacillus* species. In addition, it is considered that certain alkaliphilic* Bacillus* species produced a large amount of membrane-bound cytochrome* c* on the outer surface of membrane to support the electron transfer from the outer surface of the membrane to the inner membrane direction under a large transmembrane Δ*ψ* [29]. Furthermore, cytochrome* c* may play a role in regulating H^+^ transfer on the outer surface of the membrane via a surface protein function, sensing of Δ*ψ* and H^+^ attraction by retaining electrons in H^+^ and as an electron condenser constructed on the membrane.

**Figure 8:**
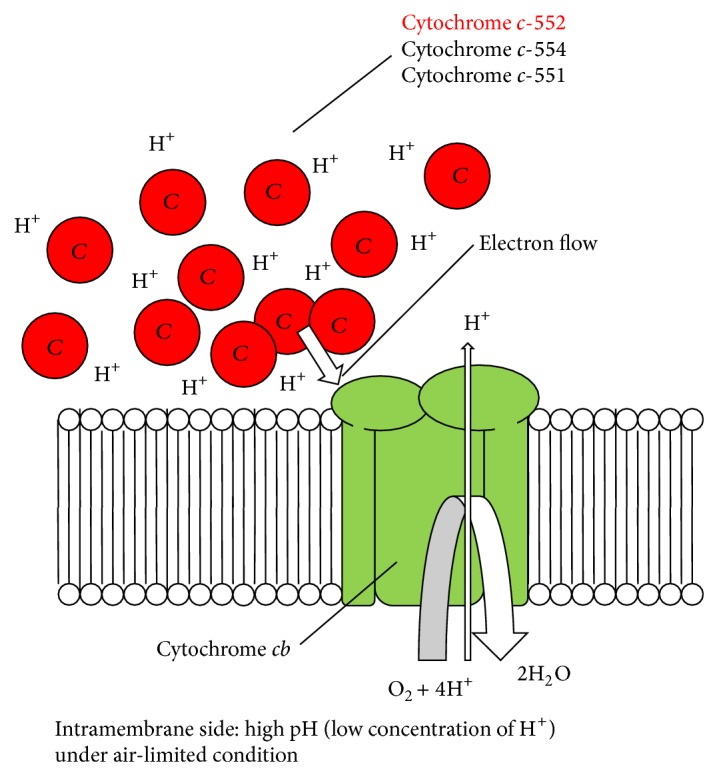
Hypothetical model of cytochrome* c*-552 function in respiratory system of* P*.* alcaliphila* AL15-21^T^. The function of cytochrome* c*-552 as an electron and H^+^ reserve enhances the function of terminal oxidation (cytochrome* cb*) at pH 10 under low-aeration intensity (H^+^-less and O_2_-less condition). Owing to the large negative charge of electron, H^+^ is attracted by the reduced cytochrome* c*-552. Thus, owing to the large electron and H^+^ capacity of enhanced amount of cytochrome* c*-552, the cytochrome* cb* terminal oxidase can transfer electrons and F_1_F_0_-ATP synthase can translocate H^+^ at pH 10 under low-aeration intensity. The hypothetical model shows that cytochrome* c*-552 in large capacity in the periplasmic space plays the role of an “electron and H^+^ condenser” in terminal oxidation and F_1_F_0_-ATP synthase activation.

On the other hand, although the contribution of Δ*ψ* in alkaliphilic* Pseudomonas* species is unknown, these bacteria utilize cytochrome* c*, which exhibits excellent electron retaining ability and a vast periplasmic space for not only accumulating H^+^ to protect against an extracellular alkaline environment but also as an H^+^ and electron condenser (Figure 8). Thus, the differences in bioenergetic alkali-adaptation strategy between Gram-positive and Gram-negative bacteria using a function of H^+^ and electron condenser related to the function of cytochrome* c* are attributed to the membrane surface structure. Owing to the phylogenic diversity of alkaliphilic* Bacillus* species, diverse variations in alkaline adaptation mechanisms probably exist in this Gram-negative bacterial group. In the near future, numerous alkaliphilic* Pseudomonas* species will be discovered. It is expected that variations in alkaline adaptation mechanisms exist among alkaliphilic* Pseudomonas *species.
